# Genomics Reveal Population Crash and Range Contraction in Elusive Fijian *Hylaeus* Bees

**DOI:** 10.1002/ece3.74366

**Published:** 2026-10-05

**Authors:** Tegan A. Edwards, Damien Esquerré, Nikolas P. Johnston, Stephen Galvin, Nikkita L. Maxwell, Francisco Encinas‐Viso, James B. Dorey

**Affiliations:** ^1^ Environmental Futures Research Centre, School of Science University of Wollongong Wollongong New South Wales Australia; ^2^ Molecular Horizons Research Institute, School of Science University of Wollongong Wollongong New South Wales Australia; ^3^ The University of the South Pacific Suva Fiji; ^4^ Centre for Australian National Biodiversity Research CSIRO National Research Canberra Australian Capital Territory Australia; ^5^ Collections Australia, CSIRO, Black Mountain Science and Innovation Park Canberra Australian Capital Territory Australia; ^6^ College of Science and Engineering Flinders University Bedford Park South Australia Australia

**Keywords:** canopy pollinator, demographic reconstruction, habitat fragmentation, island endemics, sampling methods

## Abstract

The conservation of pollinators is of particular interest due to their keystone role in ecosystem function, but is hindered by systematic geographic and methodological biases such that baseline data are not often readily available. Islands are useful as simplified systems to understand evolutionary processes, are known to host higher bee species richness per unit area than mainland regions, and have disproportionate representation of threatened species. We used low‐lying and canopy sampling paired with mitochondrial and genomic data to assess a recently discovered clade of canopy‐specialist Fijian *Hylaeus* bees. We found inter‐island genetic structure—or incipient speciation—requiring further systematic research. In the most abundant species, *H. navai* , we used genome‐wide SNPs with demographic reconstruction to infer major declines of ~99% (81.6%–99.9% most‐ to least‐conservative, respectively) in effective population size since European colonisation. Over three separate 10‐year periods in *H. navai* 's demographic history we inferred population size declines of 61%, 76% and 82%. These declines provide evidence to justify an International Union for Conservation of Nature (IUCN) assessment, with population size declines of this magnitude aligning with criteria for a listing of Critically Endangered. We found evidence of low observed heterozygosity and elevated inbreeding, consistent with a genetic bottleneck. We used species distribution models to show that *H. navai* has suitable habitat encompassing 1.4%–2.4% of Fiji's land area and is likely now restricted to high elevation forests. By examining historic collections, we raise concerns about contemporary displacement from lowland areas. We suggest that habitat modification (due to increasing human population), the invasive African tulip tree (a potential ecological trap), cyclones, and increased competition from native and introduced pollinators might all interact to drive declines. Our findings are likely not restricted to Fijian bees and provide empirical evidence of declines in hidden canopy pollinators that should be a priority for assessment across the Pacific and the world.

## Graphical abstract

We used a novel paired low‐lying and canopy sampling technique with genomic data to assess the recently discovered canopy‐specialist Fijian *Hylaeus* bee clade. We found major (~99%) declines in population size since European colonisation, limited contemporary distributions and inter‐island genetic structuring. We discuss how human population, invasive species and extreme weather are likely driving population declines in Fiji and that these forces are likely acting on other cryptic taxa more broadly.

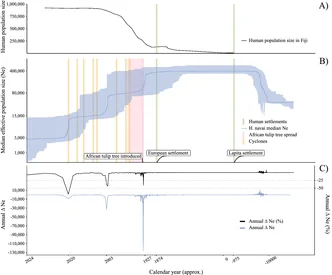

## 1 Introduction

The global decline of insects is a major issue that threatens many foundational insect‐mediated ecosystem functions, such as nutrient cycling, soil formation and decomposition, biological pest control and pollination services (van der Sluijs 2020). Despite providing innumerable essential services and comprising 60% of all animals (Forbes et al. 2018; Catalogue of Life 2025), insect conservation is comparatively under‐represented across domains (van der Sluijs 2020). For example, 15% of animals assessed by the International Union for Conservation of Nature's (IUCN) Red List are insects, a quarter of the proportional representation. Calls to face the global ‘insectageddon’ have been tempered with the need to make ‘hard‐nosed, balanced and numerical analyses’ of declines (Thomas et al. 2019).

Keystone insect groups, like pollinators, are particularly important for conservation assessments and actions because the health and stability of terrestrial ecosystems relies on them (Stephens et al. 2023). Pollinators support both wild plant diversity and agricultural productivity, underpinning food security and ecosystem services globally (Aizen et al. 2009; Garibaldi et al. 2022; Klein et al. 2006). However, mounting evidence indicates widespread pollinator declines across taxa and regions (Burkle et al. 2013; Shivanna et al. 2020; Steffan‐Dewenter et al. 2005). Global surveys and long‐term monitoring efforts have revealed considerable declines in pollinator abundance, diversity and distribution (Shivanna et al. 2020). Anthropogenic landscape change and pesticide use have been identified as major drivers, causing direct mortality and habitat loss (Dicks et al. 2021; Kevan 1975; Senapathi et al. 2017). However, many declines remain undetected, particularly in understudied regions and for cryptic or elusive taxa, thus limiting effective conservation action.

Bees (Hymenoptera: Anthophila) are the primary animal pollinators in most ecosystems, contributing to the reproduction of 78%–94% of all plant species (Danforth 2007; Ollerton et al. 2011). Since their evolution of pollen‐based diets (Danforth et al. 2013; Peters et al. 2017), bees have diversified remarkably, with a current species richness of 21,000 described species across seven families (Ascher and Pickering 2020), and an estimated richness of at least 24,705–26,164 species (Dorey et al. 2026). As with pollinators more generally, bees are thought to be experiencing widespread declines, placing many species at increasing risk of extinction (Burkle et al. 2013; Shivanna et al. 2020; Steffan‐Dewenter et al. 2005). However, only 2.6% (541/21,000) of bee species have been assessed with sufficient data on the IUCN Red List. Understanding and conserving bee species is complicated by pervasive biases in both geographic focus and sampling methods. Research remains heavily skewed towards North America and Western Europe, with tropical regions—despite their high diversity—remaining severely understudied (Orr et al. 2021; Prado et al. 2017; Skaldina and Blande 2025).

Island ecosystems represent a major opportunity for pollinator conservation in simplified ecological and evolutionary systems. Yet, despite being biodiversity hotspots with disproportionately high endemism and relatively high bee species richness (Dorey et al. 2026; Hrdina and Romportl 2017), they are among the least studied systems for bee diversity and population dynamics (Groom and Schwarz 2011). These combined biases mean that declines in many taxa likely remain undetected. Additional to geographic biases, traditional sampling methods systematically miss entire pollinator communities and there is a growing awareness, and urgency, to assess forest and canopy pollination systems (Ulyshen et al. 2023).

The Fijian archipelago is becoming a model system for understanding ecological, evolutionary and conservation issues using molecular techniques and endemic bee species (Dorey et al. 2020, 2021; Groom et al. 2013; Slattery et al. 2025, 2026). Research on the native ground‐nesting bee genus, *Lasioglossum* (Halictidae)—comprising 28 known species—has provided greater understanding of speciation processes (Dorey et al. 2020; Groom et al. 2013), pollination systems (Draper et al. 2021; Groutsch et al. 2019; Naaz et al. 2022), and has even uncovered drastic population increases in response to ancient human modification of the environment (Clark and Anderson 2009; Dorey et al. 2020; Groom et al. 2013; Slattery et al. 2026). However, an entire endemic radiation of six canopy‐specialist Fijian *Hylaeus* (Colletidae) was unknown until underrepresented canopy sampling methods were routinely implemented (Dorey et al. 2024).

This discovery shed light on the long‐standing mystery about the origin of the isolated French Polynesian *Hylaeus tuamotuensis* (Michener 1965), by describing congeners and providing a dispersal route from Australia via the Pacific. It also highlighted that the cavity‐nesting *Hylaeus* are excellent dispersers (leaving Australia at least four times) that are likely widespread and cryptic across the Pacific (Dorey et al. 2024). The small‐bodied Fijian *Hylaeus* (*μ* = 3.9 mm; Dorey et al. 2024) carry pollen internally and appear to exhibit specialisation on red‐flower canopy plants, challenging historical assumptions that bees cannot see red (Dorey et al. 2024; Prete 2004). As probable canopy/forest specialists with potentially limited foraging ranges due to their small body size (Gathmann and Tscharntke 2002), these taxa are likely vulnerable to habitat change. Dorey et al. (2024) speculated that ancient and post‐European habitat modification (Clark and Anderson 2009; Roos et al. 2016) and cyclone‐induced damage to forest fragments might have impacted populations. Additionally, the invasive African tulip tree ( *Spathodea campanulata* ) is abundant across Fiji's main island of Viti Levu, particularly in the lowlands (78% of Fiji's forests are lowland; Department of Environment Fiji 2014), and produces nectar that is toxic to honey bees and stingless bees (Brown and Daigneault 2014; Queiroz et al. 2014; Trigo and dos Santos 2000). Hence, as a red‐flowering canopy tree it could act as an ecological trap for the Fijian *Hylaeus*, while also competing with native food plants.

Canopy specialist *Hylaeus* are likely present, and cryptic, across the Australian‐French Polynesian dispersal route across the Pacific (Dorey et al. 2024). Forest pollinators are also important, under‐sampled and likely common throughout the world (Dorey et al. 2024; Ulyshen et al. 2023). Researching how the Fijian *Hylaeus* have responded to past changes, in a simplified island system, will have major implications for relatives across the Pacific and for forest pollinators more generally.

Genome‐wide coalescent‐based estimates of effective population size represent our best, and perhaps only, avenue for assessing changes in past population size for these specialist *Hylaeus* where past monitoring is impossible and present monitoring is impractical (da Fonseca et al. 2016; Ellegren 2014). These methods are increasingly implemented for flagging populations of conservation concern (Hohenlohe et al. 2021) and have been demonstrated to correlate with IUCN listings (Li et al. 2026). While demographic reconstruction estimates are not direct measures of census population sizes or extinction risk (Walcott et al. 2026; Wang et al. 2016), they are a measure of an idealised population that would experience genetic drift at that same rate (Waples 2022). Because Fiji is a simplified island system, within‐island analyses minimise the potential impact of gene flow and ecosystem boundaries (violations of panmixis). Hence, island populations should provide better approximations of idealised populations—and better estimates of effective population size—than mainland congeners. In this study, we integrate systematic randomised canopy‐focused sampling, opportunistic floral surveys, population genomics and species distribution modelling to assess the conservation status and demographic history of Fijian *Hylaeus* bees. Using genome‐wide single nucleotide polymorphisms (SNPs) from 94 specimens and mitochondrial cytochrome oxidase I (COI) from 16 specimens across four endemic species, we attempt to: (1) clarify species boundaries using mitochondrial and nuclear markers, (2) assess population genetic structure and connectivity across fragmented forests, (3) reconstruct demographic history using coalescent methods, (4) characterise genetic diversity and indicators of population health and (5) model ecological niche breadth and predict contemporary habitat suitability. As genomic sampling was uneven across species, with *H. navai* comprising the majority of sequenced specimens (*n* = 85/94), our population level analyses focus primarily on this species, while species boundaries analyses incorporate all sequenced individuals. We firstly hypothesise that population structure of Fijian *Hylaeus* species will reflect the remaining forest patch structure with minimal connectivity. We secondly hypothesise that effective population sizes will have decreased towards the present due to habitat loss and fragmentation. Beyond providing critical conservation baselines for the Fijian *Hylaeus*, this integrative approach offers a transferrable framework for detecting cryptic declines in understudied ecosystems globally, particularly in environments where traditional methods systematically miss key biodiversity.

## 2 Materials and Methods

### 2.1 Sampling Design and Specimen Handling

We built an a priori stratified‐randomised sampling regime using *R* version 4.4.0 (R Core Team 2024) and *RStudio* version 2024.12.1.563 (Posit team 2025). We prepared and manipulated spatial data using the *R* packages *sf* version 1.0.21 (Pebesma 2018; Pebesma and Bivand 2023), *terra* version 1.8.60 (Hijmans 2025b) and *geodata* version 0.6.2 (Hijmans, Barbosa, et al. 2024). For additional data visualisation and handling we used *dplyr* version 1.1.4 (Wickham et al. 2023), *ggplot2* version 3.5.2 (Wickham and Sievert 2016), *tidyterra* version 0.7.2 (Hernangómez 2023) and *paletteer* version 1.6.0 (Hvitfeldt 2021).

We sourced a road network shapefile for Fiji from the Humanitarian Data Exchange (Humanitarian Data Exchange 2025) and cropped it to the main island of Viti Levu. We downloaded land‐use rasters from the Pacific Data Hub (Pacific Data Hub 2023) and merged them into a single raster in *R* to determine forested/unforested areas. We downloaded a vector outline of Fiji from *rnaturalearth* version 1.1.0 (Massicotte and South 2023) in *R* for alignment with other spatial layers and digital elevation models (DEMs) using *geodata*. We stratified Viti Levu by elevational bands (0–300, 301–600, 601–900, and 901–1200 m) and land‐use categories—forested (‘trees’ and ‘mangroves’) or unforested (all other categories). Along Fiji's public road network, we generated ten random sampling sites in each of the three lower elevational bands. This was reduced to six sites for the 901–1200 m band, as the road network was insufficient to fill all ten sites. We ran this process twice to create a network of 36 primary sites, and for instances where those sites were inaccessible, 36 auxiliary sites. Each site was a 400 m^2^ pixel that defined the boundaries of our sampling. Our sampling map was exported as a GeoTIFF, which was accessed and annotated in the field using the *Avenza Maps* (Avenza Systems Inc. 2024) mobile application. We conducted fieldwork between April 14th and May 11th of 2025, and we used *Avenza Maps* to locate sites and record coordinates, coordinate accuracy and elevation. Our data were later verified using Google Earth satellite imagery (Google 2024).

To supplement the 900–1200 m sites and create any above 1200 m, we added sites that were only accessible by foot (Mt. Tomanivi and Mt. Batilamu) or with special permission (Monasavu Dam). Additionally, and because the Fijian *Hylaeus* are so poorly understood, we undertook further targeted collections to identify potential host plants outside of this network. We compiled the randomised and supplementary sites and treated them equally in downstream analyses. As the lower accessibility sites likely correlate with higher elevation, this may introduce sampling bias into habitat predictions, and therefore our results should be interpreted with this limitation in mind. Each randomised site was sampled by three to five people, where at least one person was always only sweep‐netting low‐lying or canopy vegetation, including native and introduced plants (~1.5 m net), and at least one person was always only sampling using a canopy net (9–14 m net). In this way, each site was sampled using both standard (low‐lying) and underrepresented (canopy) methods.

We collected specimens into vials containing absolute ethanol and stored them at < 0°C within 24 h to preserve DNA integrity (Safarikova et al. 2021). Following our return to Australia, a male and female representative for each morphotype per sampling site (*n* = 16) were pinned and identified following Dorey et al. (2024). We removed the left hind leg of each specimen—as well as the left mid leg for pinned specimens—for genetic analysis and placed them in storage at ~4°C.

### 2.2 Sequencing and Specimen Selection

To confirm morphological species assignment of our samples, we compared COI sequences from our specimens with those in Dorey et al. (2024). We sent the mid legs of the 16 pinned specimens to the Centre for Biodiversity Analysis at the Australian National University, Canberra, ACT, for DNA extraction and sequencing of COI. Rapid sequencing and the custom‐pipeline *ONTbarcoding 2.0* developed by Srivathsan et al. (2021) were used. Briefly, DNA was extracted using 10–15 μL of HotSHOT, and a short region of COI (~300 bp) was amplified using custom polymerase chain reaction (PCR) primers containing both a unique sample barcode and the gene primer. Following amplification, all PCR products were pooled and cleaned via bead purification. These PCR products were then prepared as MinION libraries using a ligation‐based kit with 200 ng of input DNA. Following sequencing, base pairs were called using *Guppy* version 4.2.3 using the high‐accuracy model (Oxford Nanopore Technologies 2020). Sequence data were then demultiplexed and barcodes reconstructed using the software *ONTbarcorder 2* (Srivathsan et al. 2021).

Of the 101 *Hylaeus* specimens collected in this study, we selected 94 for genomic analysis (85 *H. navai* , five *H. veli*, three *H. derectus* and one *H. albaeus*). We excluded six male specimens of the largest population of *H. navai* , firstly to prioritise species diversity and secondly, because males have less genetic information due to haplodiploidy. We also excluded one male *H. veli* specimen, as it was identified after the genotyping plate had been submitted. Genomic data were generated through Diversity Arrays Technologies—DArT (Diversity Arrays Technology 2023), with methods detailed in Kilian et al. (2012) and Georges et al. (2018). Briefly, genomic DNA was digested using restriction enzymes optimised by DArT. These were sequenced on an Illumina short‐read platform to obtain SNP data. The raw reads were processed using the DArTSeq bioinformatics pipeline, which performs filtering and variant calling to generate the SNP matrix (Georges et al. 2018).

### 2.3 COI Analysis

We aligned our 313 bp COI sequences to the 654 bp COI sequences from Dorey et al. (2024) using *Geneious* version 11.1.5 (Geneious 2018). We ran a phylogenetic analysis using Bayesian inference in *BEAST2* version 2.7.7 (Bouckaert et al. 2019), using *BEAUti* version 2.7.7 to produce xml files (Bouckaert et al. 2019). Firstly, we ran *bModelTest* version 1.2.1 (Bouckaert and Drummond 2017) using the whole COI sequence as a single partition for all ‘TransitionTransversionSplit’ models using *coupledMCMC* version 1.0.2 (Müller and Bouckaert 2020). Secondly, we ran the model with a fixed clock and a BirthDeath model for 100 million iterations using four heated chains, and a delta of 0.1. We found that a 123,454 substitution model with Γ rates and invariant sites was most appropriate.

Using this model configuration, we performed four independent runs in *BEAST2* to adequately explore the phylospace and checked convergence using *Tracer* version 1.7.1 (effective sample sizes > 200; Rambaut et al. 2018). We used *TreeAnnotator* version 2.7.7 (Bouckaert et al. 2019) to extract the maximum credibility tree using common ancestor heights and visualised the tree in *FigTree* version 1.4.4 (Rambaut 2018).

### 2.4 SNP Analysis

#### 2.4.1 Filtering Protocol

We created a genlight object using *dartR.base* version 1.0.5 (Gruber et al. 2018; Mijangos et al. 2022) from the DArT data and a metrics file containing specimen information including geographic coordinates, sex and morphological species assignment. We filtered, visualised and checked our SNP data using *R*, and functions from the *dartRverse* suite (Gruber et al. 2018; Mijangos et al. 2022) and *adegenet* version 2.3.11 (Jombart 2008; Jombart and Ahmed 2011). Initial SNP calling across all species (*n* = 94) produced 23,545 SNP loci, with call rates ranging from 0.20 to 1.00 (median = 0.81) and 25.5% missing data across the matrix. Average read depth per locus was 49.16 reads (median = 19.60, range = 2.50–721.90). Individual call rates ranged from 0.11 to 0.88 (median = 0.81). We selected the below filtering steps and found that stricter filtering resulted in substantial loss of loci but did not result in major changes to the results. We removed loci with call rates below 0.90, retaining 4864 loci (locus call rate range = 0.90–1.00, median = 0.94, missing data = 6.5%). We then applied a minor allele frequency (MAF) filter to exclude singletons (1/2*n), retaining 4861 loci. Finally, we randomly selected among secondary SNPs on the same sequence tag to minimise linkage bias, resulting in a final dataset of 4092 loci. Because all retained loci were polymorphic, we did not apply monomorphic filtering. All loci had 100% reproducibility and therefore no reproducibility filtering was applied. We then assessed individual coverage but excluded filtering based on call rate as uneven species representation meant it would remove species with lower coverage. The final multi‐species dataset contained 4092 loci, with call rates of 0.90–1.00 (median = 0.94) and a missing data rate of 6.5%.

#### 2.4.2 Genetic Structure and Phylogenetics

We assessed genetic structure using a principal component analysis (PCA) and a discriminant analysis of principal components (DAPC) with *dartR.base* and *adegenet*. We explored the optimal numbers of principal components and assessed genetic clustering. This led to the re‐identification of one individual, which was then confirmed using morphology. We explored species‐level relationships with phylogenetics using neighbour‐joining and UPGMA tree methods in *dartR.base*, and used *ade4* version 1.7.23 (Bougeard and Dray 2018; Chessel et al. 2004; Dray et al. 2007; Dray and Dufour 2007; Thioulouse et al. 2018), *adegenet*, and *ggplot2* to visualise the outputs of these analyses.

#### 2.4.3 Population Structure and Demography of *H. navai*

As *H. navai* comprised most of our samples (*n* = 85/94), we filtered our genlight object to contain only that species, retaining 23,545 loci (locus call rate range = 0.13–1.00, median = 0.87, 20.0% missing data, individual call rate range = 0.53–0.88, median = 0.82). We applied the same filtering protocol outlined in section 2.4.1, with the addition of filtering by individual call rate. After removing loci with call rates below 0.90, we retained 6097 loci (locus call rate range = 0.91–1.00, median = 0.97, 4.3% missing data). We then removed individuals with call rates less than 0.90, retaining 77 individuals and 6097 loci (individual call rate range = 0.92–1.00, median = 0.98, 2.8% missing data). We applied the MAF filter to exclude singletons, retaining 5883 loci, and removed secondary SNPs on the same sequence tag to minimise linkage bias, retaining 4852 loci. A final call rate filter of 0.90 was applied, resulting in a dataset of 4839 loci across 77 individuals, with a locus call rate range of 0.91–1.00 (median = 0.97) and 2.7% missing data. To examine genetic structure within *H. navai* , we ran both PCA and DAPC analyses on this dataset, including colouring populations by locality to identify potential geographic patterns.

To examine contemporary effective population size and past demography, we undertook two analyses. Firstly, we used *NeEstimator2* version 2.1 (Do et al. 2014) to estimate the contemporary effective population size of *H. navai* . Secondly, we inferred historical changes in effective population size using *Stairway Plot 2* version 2.1.3 (Liu and Fu 2020), restricting this analysis to *H. navai* as it was the only population with over 50 individuals (Drummond et al. 2005). To calibrate the timing (*x*‐axis) of our analysis, we used data from related taxa. With mutation‐rate estimates only available for two bee species— *Apis mellifera* and *Bombus terrestris* —we chose to use the former as both species are equally as distant in lineage, and in *A. mellifera*, the European honey bee, body size is more comparable to that of Fijian *Hylaeus*. We used mutation rates from Wallberg et al.'s (2014) genome‐wide SNP estimate of *μ* = 5.27*10^−9^. We used a generation time of six per year based on the nest initiation‐to‐adult emergence duration average of 37.6 days recorded for the Hawaiian species *Hylaeus anthracinus* (Magnacca and Danforth 2006; Plentovich et al. 2021). Observations of *Hylaeus bisinuatus* suggest that females of the genus can mate and initiate new nests within 24–48 h of emergence (Torchio 1984). This indicates that post‐emergence mating is rapid and, allowing a period of a few weeks to produce new nests, equates to a generation time of ~2 months. Assuming year‐round reproduction, this is consistent with other tropical bee species (Michener 2007). Although similar latitudes of Fiji (approximately −17°) and Hawaii (approximately 19°) suggest comparable climatic conditions, we applied this estimate cautiously, as actual generation times may vary due to localised environmental factors. To examine the sensitivity of our analyses to changes in generation time, we ran our analyses using generation times of 1–6 per year.

#### 2.4.4 Female‐Only *H. navai* Analyses

To assess genetic diversity, and account for haplodiploidy sex determination in bees, we generated a dataset containing only female *H. navai* specimens (*n* = 64 before filtering, 23,545 loci, locus call rate range = 0.13–1.00, median = 0.89, 18.7% missing data, individual call rate range = 0.53–0.88, median = 0.84). We applied the filtering protocol set out in section 2.4.1. After removing loci with call rates below 0.90, we retained 6146 loci (locus call rate range = 0.91–1.00, median = 0.97, 4.1% missing data). We then removed individuals with call rates less than 0.90, retaining 59 individuals and 6146 loci (individual call rate range = 0.92–1.00, median = 0.98, 2.7% missing data). We retained 5974 loci after applying the MAF filter to exclude singletons, and 4885 loci after removing secondary SNPs on the same sequence tag. We applied a final call rate filter of 0.90, yielding a dataset of 4839 loci across 59 individuals, with locus call rates of 0.92–1.00 (median = 0.98) and 2.6% missing data. We assessed genetic diversity through observed (*H*o) and expected (*H*e) heterozygosity in addition to the inbreeding coefficients (*F*_IS_) using *dartR.base*.

We used isolation‐by‐distance analyses to assess geographic population structuring through a Mantel test using *ade4*. To further verify these results, we estimated individual ancestry coefficients using the *R* package *tess3r* version 1.1.0 (Caye and François 2016). Incorporating sampling coordinates alongside genetic data, we tested values of *K* (number of ancestral populations) ranging from one to 12, with 10 replicate runs per *K* value. We used a maximum of 1000 iterations under the projected least‐squares method, with a tolerance of 10^−7^. We assessed the optimal *K* using cross‐validation scores, where lower values indicate better fit when the curve plateaus (Caye and François 2016). To test for indicators of demographic changes, we calculated Tajima's *D* using *dartR.popgen* version 1.0.5 (Gruber et al. 2018; Mijangos et al. 2022) with significance assessed using 10,000 neutrally evolving population simulations using *ms* version 2007‐10‐16 (Hudson 2002). Finally, to check that there were no differences due to ploidy, we re‐ran our stairway plots and assessed the historical effective population size for this dataset.

### 2.5 Species Distribution Modelling

We conducted species distribution modelling (SDM) using the maximum entropy (MaxEnt) approach in *R* with *dismo* version 1.3.16 (Hijmans, Phillips, et al. 2024). We downloaded WorldClim climate variables using *geodata*, with values manipulated using *terra*, *raster* version 3.6–32 (Hijmans 2025a), *sf* and *stringr* version 1.5.1 (Wickham 2023). To correct for the meridian split (180° longitudinal), we separated and rejoined climatic data before converting them into BioClim variables and the Fiji 1986 (EPSG:3460) coordinate system. We resampled the BioClim, land‐use and DEM rasters to the lowest resolution and stacked them into a single object. We assessed correlation among predictor variables in *R* with *caret* version 7.0.1 (Kuhn 2008) and removed variables with correlations > 0.9. The final seven predictors included were (1) land‐use, (2) elevation, (3) mean diurnal range, (4) isothermality, (5) max temperature of warmest month, (6) precipitation of wettest month and (7) precipitation seasonality.

As species boundaries between *H. apertus* and *H. navai* are uncertain and they likely diverged at the last glacial maxima (see below), we examined two datasets. Dataset one, *H. navai‐apertus*, includes 2025 and historical ( *H. apertus* —22 specimens from Taveuni in 2019—and *H. navai* —2 × 2002 in Taveuni, 1 × 2003 and 3 × 2018–2019 Viti Levu) records. This dataset acknowledges that, even if these species are completely isolated, their recent divergence suggests that they are likely ecologically similar. Dataset two, *H. navai* ‐only, provides a more conservative assessment of present‐day habitat associations. Here, we chose not to use the historical dataset for *H. navai* (three *H. navai* specimens from 2002 and 2003; 140–1064 m) to both account for range contraction and to remove any bias from potential historical misidentifications.

We loaded occurrence points into *R* and generated 10,000 random background points for the SDM using *dismo*. We separated the occurrences into testing and training records using 5‐fold cross‐validation and built the model using background points, test coordinates, and the selected environmental variables. We assessed model performance and determined the optimal threshold to maximise specificity and sensitivity. We used *ggplot2*, *ggspatial* version 1.1.10 (Dunnington 2025) and *tidyterra* to visualise the outputs of the models. This same method was replicated with the occurrence data for the *H. navai* ‐only collections.

We downloaded occurrence records for the invasive African tulip tree from the Global Biodiversity Information Facility (GBIF) using *dismo* (Global Biodiversity Information Facility 2023). We then repeated the above modelling process for the African tulip tree to evaluate potential overlap in suitable habitat with the endemic *Hylaeus* species.

## 3 Results

### 3.1 Sampling Results

We sampled a total of 54 locations across Viti Levu, Fiji (Figure 1); 29 primary sites, five auxiliary sites and 20 supplemented sites (e.g., Mt. Tominivi, Mt. Batilamu and areas surrounding the Monasavu Dam). Of the 34 randomly generated sites, 20 were classified as forested while 14 were unforested. Three of the planned primary sites were excluded due to inaccessibility, safety concerns, or where permission to sample could not be attained. In these cases, auxiliary sites were chosen to maintain sampling coverage.

**FIGURE 1:**
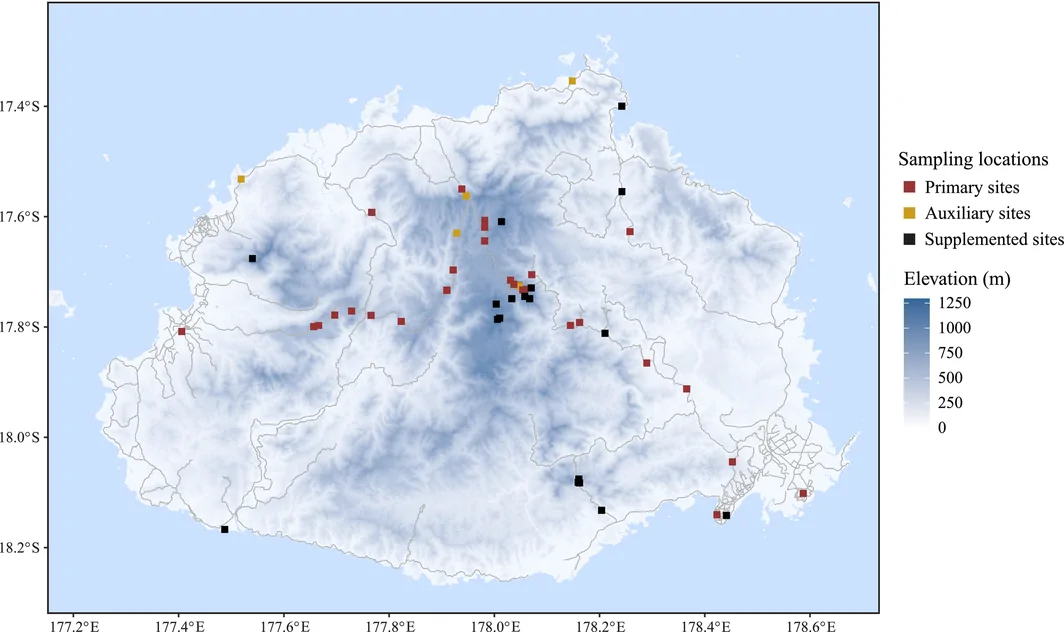
Sampling locations visited across Viti Levu, Fiji. Square points represent primary, auxiliary and supplemented sampling locations, overlayed on a digital elevation model (DEM) to illustrate topographic variation. The road network used for site selection is shown in grey.

We collected a total of 101 *Hylaeus* specimens, from a variety of plant species dominated by *Geissois superba* and *Metrosideros collina* (Table 1). We collected *Hylaeus* specimens across Viti Levu at elevations ranging from 371 to 1334 m.

**TABLE 1:** Number of *Hylaeus* specimens collected in Fiji, including those found prior to 2019 before description in Dorey et al. (2024), with elevation ranges and floral associations observed.

Species	No. specimens		Elevation range (m)		Floral associations
	1970–2019	2025	1970–2019	2025	
*H. navai*	6	91	140–1072	883–1334	*Geissois superba* (87), *Trema micranthum* (3), *Decaisnina forsteriana* (3), Unknown (Malaise) (3)
*H. veli*	3	6	875	1334	*Metrosideros collina* (10)
*H. derectus*	8	3	898–904	371–900	*Decaisnina forsteriana* (8), *Commersonia bartamia* (1), Myrtaceae sp. (2)
*H. albaeus*	12	1	3–600	923	Myrtaceae sp. (1), *Metrosideros collina* (1), Unknown (Malaise) (11)
*H. apertus*	22	—	875	—	*Metrosideros collina* (22)
*H. breviflavus*	1	—	500–600	—	Unknown (1)
Total	52	101			

### 3.2 Genetic Structure and Phylogenetics

The PCA of genome‐wide SNPs showed clear differentiation between the species collected on Viti Levu, with the first two PCs explaining most of the variation in the data (PC1 = 46% and PC2 = 18%; Figure 2A). As our SNP dataset was generated exclusively from Viti Levu specimens collected in 2025, this analysis reflects the genetic diversity within the single island and does not include comparisons with Taveuni populations.

**FIGURE 2:**
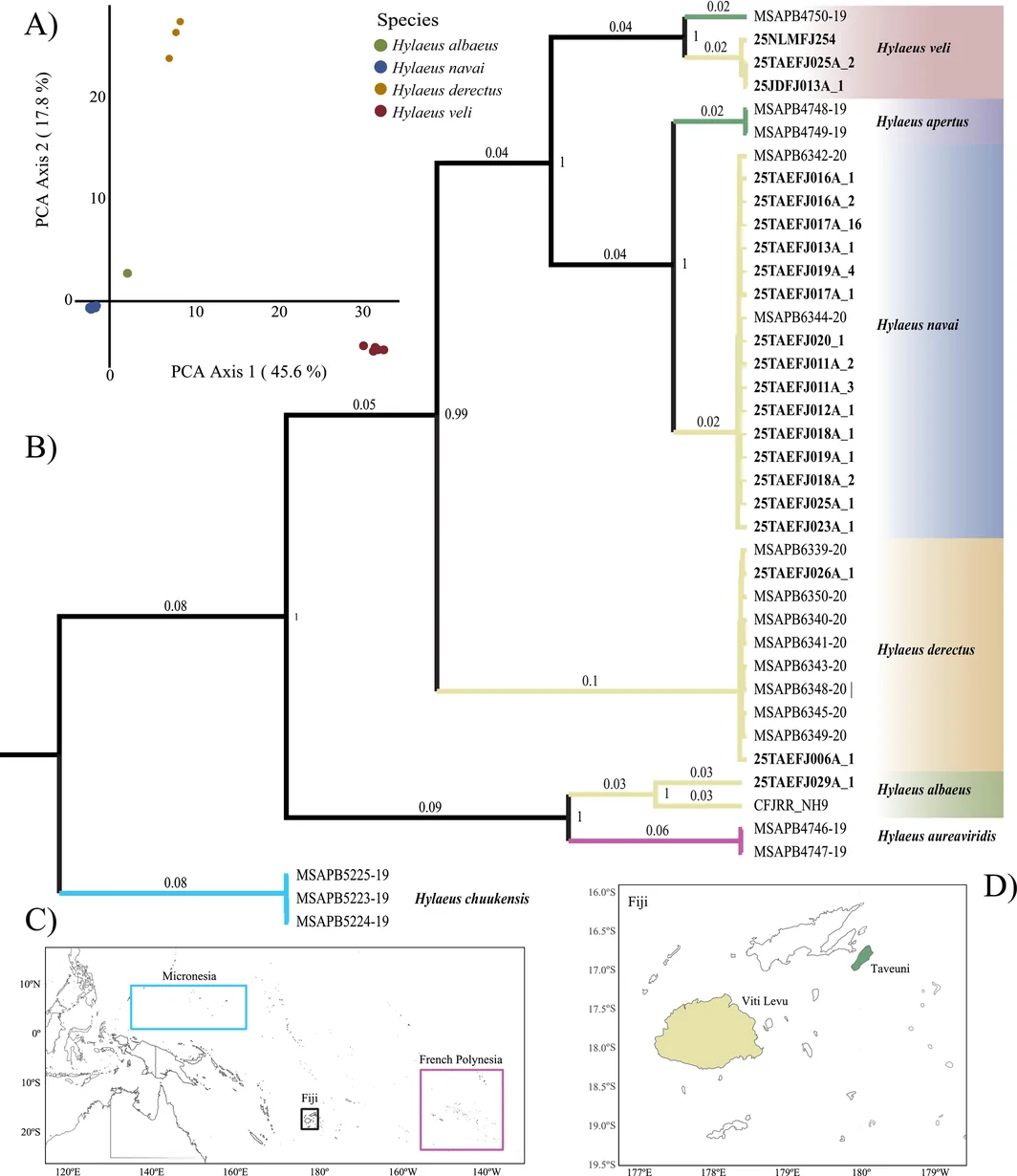
Phylogenetic relationships of Fijian *Hylaeus* species. (A) A principal component analysis of single nucleotide polymorphism data illustrating nuclear differentiation among species. (B) A COI phylogeny of historical and contemporary (from this study; bolded) specimens, with branch lengths and node support (posterior probabilities) indicated; node colours correspond to sampling locations as indicated in (C) and (D). (C) A map showing the geographic context of Micronesia, Fiji and French Polynesia. (D) A map of Fiji with sampling localities of Viti Levu and Taveuni highlighted.

To assess inter‐island genetic divergence, we relied on our COI phylogeny to compare specimens previously collected data on Viti Levu and Taveuni islands. Our Bayesian COI phylogeny had a well‐resolved topology with strong support based on posterior probabilities at all major nodes (Figure 2B). We present the first sequences of *H. veli* from Viti Levu, where historically it is only known to occur on Taveuni, and the first male specimens of *H. navai* . The branch lengths between the Viti Levu and Taveuni clades of *H. navai* and *H. apertus* are almost equal to those for *H. veli* on the two islands. We also found a deeper node between the two *H. albaeus* specimens than that found between *H. navai* and *H. apertus* or between the two island populations of *H. veli*.

### 3.3 Population Genetics of *H. navai*

Our analyses of the female‐only *H. navai* SNP dataset indicated little evidence of population structuring. The PCA explained 3.3% and 3.1% of the variance along principal components (PCs) one and two, respectively (Figure S1). Additionally, our DAPC identified the most likely number of populations as one. The cross‐validation scores from the *tess3r* analysis failed to plateau, gradually declining across all tested *K* values, indicating limited population structuring (Figure S3). Observed heterozygosity among females was significantly lower than expected heterozygosity across loci (*H*o = 0.08, *H*e = 0.10; paired *t*‐test: *t* = −32.57, df = 4.84, *p* < 0.001). The mean difference of −0.02 between *H*o and *H*e indicates a heterozygote deficit, consistent with a positive inbreeding coefficient (*F*_IS_ = 0.18). Our Mantel test showed no significant correlation between genetic and geographic distances (*p* = 0.56), indicating no evidence of isolation by distance (Figure S4). Tajima's *D* was negative, but not significantly so, suggesting a possible weak excess of rare alleles (*D* = −1.55, *p* = 0.08). Stairway plots run under the same assumptions as the full dataset closely matched the timings and patterns of the full dataset.

The contemporary effective population size (*N*e) estimates from *NeEstimator2*—which were not corrected using mutation rate or generation time—varied with allele frequency thresholds. At a minimum allele frequency of 0.05, contemporary effective population size was *N*e = 121 (95% CI = 119, 124). Excluding only singleton alleles gave a contemporary effective population size of *N*e = 222 (95% CI = 188, 192), while including all alleles (minimum allele frequency threshold = 0+) increased contemporary effective population size further to *N*e = 418 (95% CI = 178, 181). This pattern of increasing effective population estimations with progressively more rare alleles retained in the analysis is consistent with the bias introduced with low‐frequency alleles included in LD‐based effective population estimates (Waples 2024). As the most conservative method of removing bias is excluding alleles with frequency less than 0.05, we consider *N*e = 121 to be the most reliable estimate, though we note this likely comes with some loss of precision (Waples 2024). This contemporary estimate is notably lower than the final effective population size inferred from our *Stairway Plot 2* reconstruction, with no overlap of confidence intervals. This discrepancy likely reflects key methodological differences between the approaches, as *Stairway Plot 2* considers mutation‐rate and generation‐time parameters, whereas the *NeEstimator2* estimate is calculated directly from linkage disequilibrium patterns in the data. Finally, *Stairway Plot 2* is known to have issues resolving contemporary effective population size (Walcott et al. 2026), which likely further drives the inconsistencies in the estimates.

The stairway plot revealed marked changes in effective population size through time (Figure 3B). Our reconstruction suggests a population increase in the long past, which should not be interpreted as it likely reflects an artefact (Liu and Fu 2015, 2020). Sensitivity analyses using generation times of 1–6 per year showed qualitatively similar patterns of decline, with four or six generations per year matching closely with human population size expansion in Fiji. We used six generations per year, which matches the life history of the related *H. anthracinus* , a species that inhabits a similar latitude in Hawaii (Magnacca and Danforth 2006; Plentovich et al. 2021; Figure S2).

**FIGURE 3:**
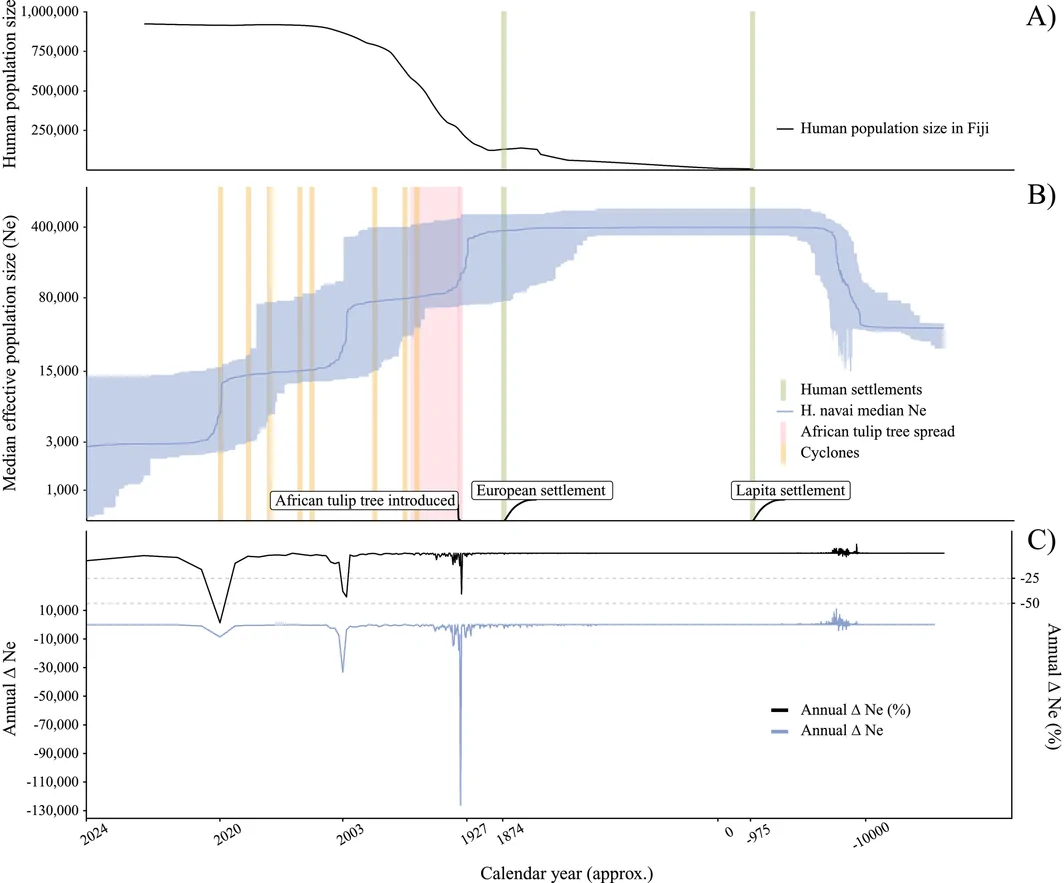
Demographic history of *H. navai* in Fiji in relation to human settlement and environmental change. (A) Human population size in Fiji over time, with green bars representing settlement events (Lapita and European colonisation). (B) Stairway plot showing effective population size (log scale) of *H. navai* through time, with 95% confidence intervals represented by blue shaded areas. Green bars represent human settlement events as in (A); yellow bars indicate major cyclones affecting Viti Levu; the pink bar represents the introduction of the African tulip tree and the subsequent theorised 40‐year period to reach carrying capacity. (C) Annual change in effective population size of *H. navai* , with the actual change shown as a blue line (left *y*‐axis) and proportional change shown as a black line (right *y*‐axis). The *x*‐axis for all plots is scaled to approximate calendar years in log scale to emphasise demographic changes.

Declines in the effective population size potentially begin around the year 1550, with a 7% drop until the late 1800's, with the lower 95% confidence interval making a steeper decline possible (Figure 3B,C). However, the confidence intervals are broad, and median declines remain small during this time. From around 1874, coinciding with European colonisation of Fiji and subsequent human population size increases (Figure 3A), declines were inferred in most years. The first major 10‐year decline (commensurate with IUCN criteria) between 1933 and 1942 saw effective population size drop by ~190,000 (−61%; Figure 3B,C). Another sharp decline occurred around 2003, reducing effective population size from ~62,000 to ~15,000 (−76%) between 2002 and 2011. A final contraction was observed around 2020, with effective population size falling from ~14,000 to ~2600 (−82%) between 2016 and 2025. It should be noted, again, that the wide and gradual confidence intervals could mask small population declines within those bounds. Therefore, 10‐year IUCN windows are more appropriate to assess population decline, as opposed to annual change data. Taken together, the sequential major declines and gradual continued declines represent a 99% reduction in effective population size over the past ~150 years, since European colonisation of Fiji (Table S1). Taking the most‐conservative (past lower and present upper 95% confidence intervals) and least‐conservative (past upper and present lower 95% confidence intervals) values for population decline, we found a range of 81.6%–99.9% decline (Table S1).

### 3.4 Species Distribution Modelling

Our initial SDM of the combined *H. navai* ‐*apertus* occurrence datasets—incorporating both historical and contemporary occurrence data—showed high predictive performance (AUC = 0.99; Figure 4A). The most important predictors of habitat suitability (Figure 4B) were max temperature of warmest month (BIO5; 47.5%), land‐use (22.2%) and elevation (22.1%). Suitable habitat (> 50% probability) was estimated to comprise 2.4% of the Fijian archipelago. The SDM based on the *H. navai* ‐only occurrence records from Viti Levu also had high predictive performance (AUC = 0.99; Figure 4C). Key predictors differed from the combined model, with isothermality (BIO3; 62.2%) and elevation (36.7%) emerging as the strongest drivers, while all other predictors had negligible importance (Figure 4D). In this model, suitable habitat was more restricted, covering 1.4% of the study area and was confined to the mid‐to‐high elevation regions. Despite the difference in size, both models identified similar regions as being suitable.

**FIGURE 4:**
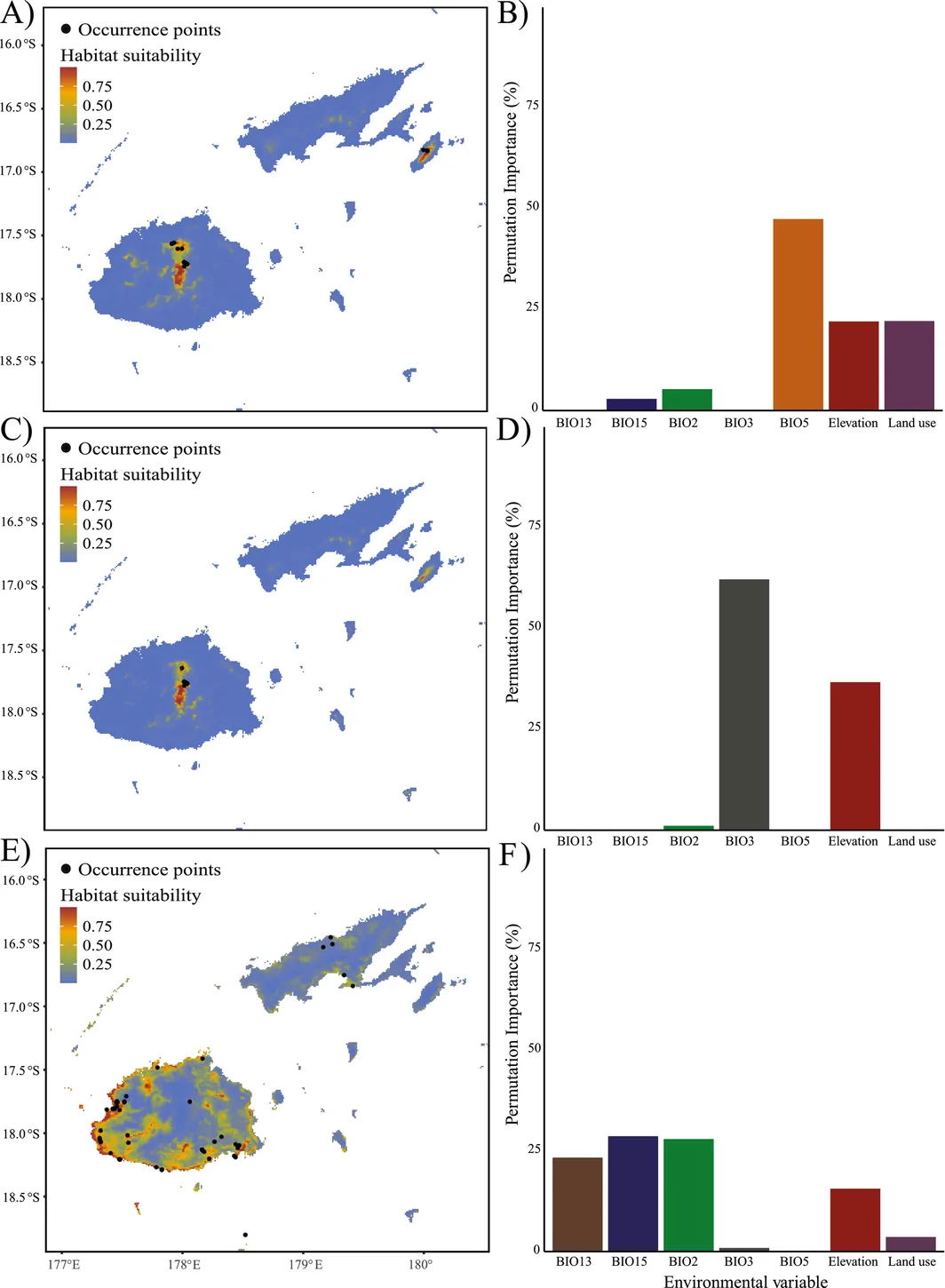
Species distribution models (SDMs) and corresponding variable importance for Fijian *Hylaeus* and African tulip trees. (A, C, E) The predicted habitat suitability for *H. navai‐apertus*, *H. navai* ‐only and African tulip tree, with black points representing occurrence locations. (B, D, F) The corresponding permutation importance variables for each of the SDMs.

The SDM for the invasive African tulip tree had lower, but still high, predictive performance (AUC = 0.88; Figure 4E). Habitat suitability for the African tulip tree was primarily driven by precipitation seasonality (BIO15; 28.6%), mean diurnal range (BIO2; 27.9%), precipitation of wettest month (BIO13; 23.3%) and elevation (15.6%; Figure 4F). In contrast to the *Hylaeus* SDMs, the African tulip tree was predicted to have a much larger extent of suitable habitat, encompassing 13.4% of the study area, concentrated at lower elevations. This range was largely inverse to the *H. navai‐apertus* SDM.

## 4 Discussion

In this study, we investigate the ecology, genetic health, population structure and distribution of the endemic Fijian *Hylaeus* bees, with a particular focus on *H. navai* —for which we collected the most specimens. Using field surveys, phylogenetic analyses, population genomics and species distribution modelling, we provide the first integrated study of the Fijian *Hylaeus*. We support previous findings of habitat and diet specialisation, and we raise questions about species boundaries, particularly between islands. Importantly, we find empirical evidence of major population declines in *H. navai* , a recently discovered species in need of major conservation action, with broader implications for undiscovered forest pollinators globally.

### 4.1 Sampling and Elevation Patterns

To improve upon traditional pollinator surveys that have been completed in Fiji and globally, we used a sampling regime that incorporated systematic sampling of both canopy and underlying vegetation with a primarily randomised sampling design. Our sampling encompassed the full elevational gradient of Viti Levu, including extensive sampling across the lowlands where *Hylaeus* were previously recorded (Dorey et al. 2024). Our approach of targeting both forest canopy (up to 15 m) and low‐lying vegetation (< 2 m) minimised the number of bees missed due to sampling bias (Ulyshen et al. 2023). Across our 54 sampling locations, we collected 101 *Hylaeus* specimens, revealing distinct elevational abundance patterns among species (Table 1). We found *H. derectus* between 371 and 900 m, suggesting a relatively broad elevational niche and potential tolerance of lowland habitats. Similarly, *H. albaeus* has now been found across a wide elevational range of 3–923 m; having previously only been recorded below 600 m (Dorey et al. 2024). In contrast, *H. navai* and *H. veli* have only been collected between 875 and 1334 m, suggesting a much narrower elevational niche. Critically, 26/54 of our randomly generated and haphazardly sampled sites were at elevations below 600 m, where we only collected a single *H. derectus* specimen. This was despite an average sampling time across all locations of 48 min per site.

These patterns could indicate species‐specific ecological specialisation to elevational niches. Comparable patterns have been observed in the endemic Fijian *Lasioglossum* (*Homalictus*) bees, where many species show narrow elevational ranges, with many high elevation specialists (Dorey et al. 2020). Hence, the Fijian *Hylaeus* could similarly comprise elevational generalists and specialists. However, the near absence of *Hylaeus* from lowland habitats in our study, where they are historically known (1970–2016), is extremely concerning and could be an indication of major declines or extirpations in lowland areas.

### 4.2 Species Boundaries and Divergence

Resolving species boundaries within the Fijian *Hylaeus* is both taxonomically complex and ecologically important, as uncertainty in delimitation restricts our ability to interpret patterns of diversity and demographic history. Our COI‐based phylogeny agreed with the topology in Dorey et al. (2024), reinforcing confidence in the overall relationships among species while also revealing new insights through expanded sampling (Figure 2B).

Across the islands of Viti Levu and Taveuni and using COI, we found similar divergences between *H. navai* and *H. apertus* as within *H. veli* (Figure 2B). This could indicate either incipient speciation events or over‐splitting of species concepts. As all the specimens sequenced for genomic SNPs were from Viti Levu, collected in 2025, we were unable to further investigate these divergences through genomics. We suggest that these clades represent valuable evolutionarily significant units (Fraser and Bernatchez 2001; Moritz 1994) and that they might represent a post glacial‐maximum split when the islands were joined ~19,000 years ago (Clark et al. 2012; Dorey et al. 2021). We note that the deep divergence within *H. albaeus* might be caused by poor sequence quality in the type specimen, or that the specimen might be *H. breviflavus*, as no females have been found.

Collectively, our results highlight the complexity of species delimitation in the Fijian *Hylaeus* and the need for future field studies prioritising sampling. Multiple lines of evidence—COI divergence, morphology and genomic analyses—are necessary to accurately delimit these species boundaries. The population genomic methods applied here to *H. navai* , including genome‐wide SNPs, coalescent‐based demographic reconstruction and diversity assessments, are directly applicable to the remaining Fijian *Hylaeus* species once sufficient samples are obtained. Two genome‐wide SNP samples can be sufficient to calculate pairwise *F*_ST_ (Nazareno et al. 2017); however, population sample sizes of ≥ 50 would also allow robust demographic reconstructions (Drummond et al. 2005). We recommend further sampling and sequencing efforts of both newly collected and museum specimens. Similar levels of cryptic diversity and inter‐island differentiation may exist across the radiation, with implications for conservation prioritisation and understanding of Pacific Island and Oceania insect biogeography more broadly (Hending 2025).

### 4.3 Population Structure and Past Demography of *H. navai*

*Hylaeus* *navai* was the most extensively sampled species in our study, with multiple collection sites across Viti Levu's highland forest. This facilitated analyses of population structure within those forests, in addition to exploring historical demography. Our analyses of genome‐wide SNPs within *H. navai* suggested no population structuring across the highland collection sites. The PCA, DAPC and tess3r analyses suggested a single population, consistent with historical connectivity (Figure S1). Isolation‐by‐distance analyses also found no significant geographic structuring, despite the species' small body size and potential dispersal limitations (Figure S4). However, we only collected *H. navai* in the contiguous highland forests, where minimal deforestation across a relatively small area likely facilitates ongoing gene flow. Hence, we could not reject—nor meaningfully address—our first hypothesis that contemporary population structure will reflect remaining forest patch structure. Nevertheless, our COI data indicated divergence between island populations, suggesting that structuring could occur at larger geographic scales. We suggest that more intensive sampling be undertaken to examine intra‐island gene flow barriers.

Because our contemporary effective population size estimates were inconsistent, we chose not to interpret them here. However, our stairway plot estimates of *H. navai* 's effective population size showed major long‐term declines (Figure 3B). While specific timings (*x*‐axis) of demographic events should be interpreted with caution, as they are influenced by assumptions about mutation rates and generation times (Liu and Fu 2015), the timing of demographic events aligns broadly with several known periods of ecological and anthropogenic change in Fiji (Figure 3A). We consider these results in relation to the hypothesis raised by Dorey et al. (2024), who stated that the Fijian *Hylaeus* would be ‘vulnerable to both ancient and contemporary clearing of forests’. The first evidence of slash‐and‐burn agriculture in Fiji is from 1.8 kya (~225 BC; Clark and Anderson 2009; Roos et al. 2016). Our earliest inferred period of population declines began around 1556 with an estimated ~7% decline in the 300 years until 1874, when European colonisation began, and coincides broadly with a period of population growth (Our World in Data 2024). Although slash‐and‐burn agriculture might not have directly caused immediate population declines, the intensive land‐use changes and creation of dry, fire‐tolerant conditions on Viti Levu could plausibly have initiated long‐term habitat changes (Clark and Anderson 2009), contributing to the demographic patterns observed here.

Our genomic data indicate that, post‐european settlement, *H. navai* populations continued to decline as human populations increased. This decline is consistent with increasing anthropogenic pressure on forest habitat. We inferred three major periods of decline that we choose to interpret in 10‐year intervals to (1) not over‐interpret the *x*‐axis and (2) to align with the 10‐year period in which IUCN declines can be assessed. These periods of decline were 1933–1942 (61% decline), 2002–2011 (76% decline) and 2016–2025 (82% decline; Figure 3B,C).

### 4.4 Species Distribution Modelling and Habitat Suitability

To complement our population genomic analyses, we used species distribution modelling (SDM) to examine environmental drivers of habitat suitability and assess potential range patterns. While genomic data reveal population connectivity and demographic trends (Steiner et al. 2013), SDMs provide a spatial perspective on where suitable habitat could occur (Miller 2010). Due to uncertainties around species boundaries, we created two *H. navai* SDMs. *Hylaeus* *navai* and *H. apertus* are closely related and, even if species boundaries are firm, they likely share similar niches. Therefore, we ran *H. navai* ‐*apertus*‐ and *H. navai* ‐only SDMs (Figure 4A,C). Because the divergence between *H. navai* and *H. apertus* is recent and Taveuni island (the current distribution for *H. apertus* ) is less modified, we employed this dual approach to examine potential impacts of anthropogenic change on species distributions.

Our SDMs showed strong predictive performance based on cross‐validation, indicating that the models effectively discriminated suitable from unsuitable habitat. Both the combined *H. navai* –*apertus* model and the *H. navai* ‐only models showed good fit (AUC = 0.99); although the predictor variables varied between them. The combined *H. navai* –*apertus* model predicted suitable habitat across 2.4% of Fiji, while the *H. navai*‐only model predicted 1.4%. Both models had suitable habitat predictions concentrated in mid‐to‐high elevation regions (Figure 4A,C). Our African tulip tree model, although somewhat less precise, still outperformed random predictions (AUC = 0.88; Figure 4E). The model predicts a much larger extent of suitable habitat (13.4%) for the African tulip tree, which is concentrated in lower elevations (Figure 4E).

While these models have not been field‐validated with independent surveys—something that should form the basis of future work—and should be interpreted as hypotheses, they are mostly consistent with our field observations. For example, we anecdotally saw African tulip trees more frequently in the lowlands. We do not know if this represents ongoing invasion dynamics where more intact highlands are resilient to invasion, if the model is detecting physiological bounds (i.e., abiotic interactions), or likely, a combination of both. However, the African tulip tree is thought to favour warmer climates and might expand its distribution with climate change (Chan 2025). The possible extent of African tulip tree expansion poses a non‐trivial threat, if it is indeed poisoning native Fijian *Hylaeus* bees.

### 4.5 Conservation Implications

The Fijian *Hylaeus* remain rare and difficult to sample, even where host plants are present. From our sampling, *H. navai* represents either the most common, easiest to catch, or most seasonally abundant *Hylaeus* species in Fiji. Despite this, *H. navai* has undergone major population declines of 99% since European colonisation and three subsequent 10‐year declines that could individually justify IUCN listing as Vulnerable (61% in 1933–1942), Endangered (76% in 2002–2011) and Critically Endangered (82% in 2016–2025; Figure 3B,C). Alarmingly, these declines do not appear to be abating and highlight how, as population sizes decline, proportional losses can increase. While declines in *H. navai* effective population size are clear and support our second hypothesis of declining populations towards the present, the exact causes are not empirically known. However, we do raise several possible, and likely interacting, drivers, worthy of future research.

Firstly, the declines are commensurate with increasing human population sizes (Figure 3A). Human population growth can burden the natural world and drive population declines (Hogue and Breon 2022). In fact, Hogue and Breon (2022) found that 88% of IUCN‐listed species were impacted by habitat destruction. In Fiji, between 1991 and 2007, closed natural forests declined by 21% and mangroves by 5%, mostly through urban and agricultural expansion (Department of Environment Fiji 2014; Roos et al. 2016). A combination of impacts including deforestation, agricultural expansion and habitat fragmentation (Kelly 1995) could disproportionately impact habitat and food specialists like the Fijian *Hylaeus*. Less disturbed islands of the Fijian archipelago, like Kadavu and Taveuni, could provide opportunities to empirically test how habitat destruction and/or the threats identified below have impacted *H. navai* populations.

The second focus of future research should concentrate on the invasive African tulip tree, which was introduced as an ornamental to Fiji in the 1930s (Smith 1979). This species is believed to reach carrying capacity roughly 40 years post‐introduction to an area (Brown and Daigneault 2014) and is, therefore, unlikely to have caused an immediate decline in bee populations. However, the subsequent progressive decline of *H. navai* could, in part, be driven by the spread of the invasive tree. We found that the *H. navai* and African tulip tree SDMs were inverse, with the former mostly modelled in the highlands and the latter in the lowlands (Figure 4A,C,E). Additionally, while 16/20 pre‐2019 Fijian *Hylaeus* occurrences were found < 800 m (Dorey et al. 2024) only 1/101 were found < 800 m in this study, a trend that occurred despite targeted and extensive sampling across Viti Levu's elevational range (Table 1). Similarly, we found that *G. superba* trees at lower elevation sites (356 and 498 m) hosted only *Lasioglossum* (*Homalictus*) bees. Currently, there are no comprehensive maps for the distribution of the African tulip tree across Fiji, but developing such maps would be valuable and can be achieved using spectral imagery (Chan 2025).

Thirdly, cyclones—known to destroy tree cover in Fiji (Metherall et al. 2025)—are a possible driver of *Hylaeus* decline in the country. Such destructive events can have a direct impact on pollinator assemblages, with reductions in species richness and diversity (Landry 2013). While the severe population decline of *H. navai* in 2020 corresponds with Tropical Cyclone Harold—a category four event in April of that year—we urge caution in this exact interpretation. It should be noted that the 2015 specimen of *H. albaeus* was collected in Rakiraki, Viti Levu and could not be re‐collected following major destruction by Tropical Cyclone Winston in 2016, despite repeated and targeted efforts in 2017, 2018, twice in 2019 and in 2025. The likelihood is that the habitat is no longer suitable for that population, or that recolonisation is non‐trivial in a fragmented landscape. The effects of human disturbance, such as reduced canopy cover and forest patch size in addition to weakened trees, will exacerbate the effects of tropical cyclones, resulting in more severe habitat damage as smaller patches become more susceptible to storm‐induced mortality (Esseen 1994; Stork and Turton 2009). The interactions between anthropogenic fragmentation and natural disturbance regimes suggest that demographic declines could accelerate in coming decades under projected increases in cyclone intensity (Knutson et al. 2020). Additionally, cyclones likely accelerate the spread of African tulip trees in the Pacific through increasing seed dispersal and creating disturbed habitats favourable for its establishment (Chan 2025).

Cyclones might also exacerbate reduced habitat availability by dwarfing forests, lowering canopy cover (Lin et al. 2020), destroying stem nests, changing microclimates and reducing suitable habitat for *Hylaeus* (U.S. Fish and Wildlife Service 2022). The potential role of canopy structure in providing suitable habitat for these species suggests that forestry practices, cyclone impacts and fragmentation effects on canopy development could drive declines in even protected forests. This has implications for conservation planning across tropical forest systems, where canopy‐dwelling species remain understudied but likely face similar constraints.

Additionally, habitat‐change driven competition and invasive species may impact the Fijian *Hylaeus*. The super‐generalist *Lasioglossum* (*Homalictus*) *fijiense*—which feeds on the same plants as the Fijian *Hylaeus*—underwent population expansions in response to ancient human land use (Dorey et al. 2021). Such changes could have increased the permeability of previously forest‐dominated areas and increased competitive pressure on *Hylaeus* for floral resources. However, it could be expected that if genetic signals persisted through the present 99% bottleneck, the impacts of *L. fijiense* competition would be detectable concurrently with those of slash‐and‐burn agriculture (Dorey et al. 2021), while multiple stressors could exacerbate increased competition. Additionally, the European honey bee, introduced to Fiji in 1874, is known to impact native pollinators (Wojcik et al. 2018), and may further reduce resource availability. While the honey bee was detected at almost every sampling site examined for this project, it was rarely in high abundance.

Thermal filtering—where average temperatures decrease with elevation (Anderson et al. 2018)—could possibly explain contemporary elevational trends. However, in the face of inferred population declines and possible withdrawals from lowland regions, this seems unlikely. Recent warming trends across Fiji (0.018°C–0.024°C increase in daily maximum per year; Sagero et al. 2025) could still narrow suitable elevational niches. Experimental work on megachilid bees showed that individuals translocated to warmer, lowland environments had reduced emergence rates—that is, the number of pupae successfully developing and emerging as adult bees—whereas those relocated to cooler, higher elevations showed increased emergence (McCabe et al. 2022). As the Fijian *Hylaeus* are likely also cavity nesters (Dorey et al. 2024), these thermal constraints might partly explain their apparent greater abundance at higher elevations. Additionally, warming climates might favour African tulip tree spread in higher elevations (Chan 2025), thus extending the potential ecological trap threat for Fijian *Hylaeus*.

## 5 Conclusions

We found robust evidence of declines in canopy‐specialist pollinators using cross‐disciplinary methods and analyses. Our study integrates systematic field surveys, population genomics and species distribution modelling to assess the conservation status and demographic history of endemic Fijian *Hylaeus* bees. We used paired low‐lying and canopy sampling to support previous implications that the Fijian *Hylaeus* are canopy and red‐flower specialists. However, we found uncertain species boundaries and inter‐island genetic differentiation, highlighting the need for expanded genomic analyses and systematic sampling efforts across the archipelago.

A striking ~99% reduction in *H. navai's* effective population size was revealed over ~150 years, coinciding with European colonisation, habitat modification and invasive species establishment. Our SDM modelling and inferences from historical and contemporary sampling suggested that *H. navai* , and the Fijian *Hylaeus* more broadly, might have contracted from lowland areas where impacts from anthropogenic modifications and invasive species, for example, African tulip trees, might be highest. Our models suggest that *H. navai* has been pushed out of lowland areas and is currently restricted to 1.4%–2.4% of Fiji's terrestrial area, mostly at higher elevations. Together, our sampling and molecular and geospatial analyses provide strong evidence for major range and population contractions. We suggest that this should form the basis of a Critically Endangered status for *H. navai* based on IUCN criterion A2b (CR A2b). Empirically identifying, and counteracting, that the major drivers of decline is of immediate conservation importance for all Fijian *Hylaeus* bees.

We suggest that the Fijian *Hylaeus* can serve as a model system for understanding past declines in cryptic taxa. We also highlight the foundational importance of systematics in identifying such cryptic taxa before they are lost to extinction. Because canopy‐specialist *Hylaeus* are expected to be widespread across the Pacific, linking Australian and French Polynesian taxa (Dorey et al. 2024), we suggest that threats to cryptic *Hylaeus* species will be widespread. We also highlight the strong possibility of cryptic canopy‐specialist pollinators globally. Conservation biologists should consider using systematic and targeted multi‐strata sampling approaches paired with molecular techniques to make powerful inferences about population dynamics and declines, achievements that are impossible using standard ecological sampling alone. Because baseline data do not exist for most taxa—especially poorly understood invertebrates—our integrative framework provides a roadmap for detecting cryptic declines and drivers in forest canopies globally.

## Author Contributions

**Tegan A. Edwards:** conceptualization (equal), data curation (lead), formal analysis (lead), methodology (equal), validation (equal), visualization (equal), writing – original draft (lead), writing – review and editing (lead). **Damien Esquerré:** conceptualization (supporting), data curation (supporting), formal analysis (supporting), funding acquisition (equal), methodology (supporting), project administration (supporting), supervision (equal), validation (equal), writing – original draft (supporting), writing – review and editing (supporting). **Nikolas P. Johnston:** conceptualization (supporting), data curation (supporting), formal analysis (supporting), funding acquisition (equal), methodology (supporting), project administration (supporting), supervision (equal), validation (equal), writing – original draft (supporting), writing – review and editing (supporting). **Stephen Galvin:** funding acquisition (supporting), methodology (supporting), project administration (supporting), resources (lead), writing – original draft (supporting), writing – review and editing (supporting). **Nikkita L. Maxwell:** data curation (supporting), methodology (supporting), writing – original draft (supporting), writing – review and editing (supporting). **Francisco Encinas‐Viso:** formal analysis (supporting), methodology (supporting), software (supporting), validation (supporting), writing – original draft (supporting), writing – review and editing (supporting). **James B. Dorey:** conceptualization (lead), data curation (equal), formal analysis (supporting), funding acquisition (lead), investigation (equal), methodology (equal), project administration (lead), resources (equal), supervision (lead), validation (supporting), visualization (supporting), writing – original draft (supporting), writing – review and editing (supporting).

## Funding

This project was funded by the Department of Foreign Affairs and Trade, Australian Government through the New Colombo Plan Grant to J.B.D., D.E., and N.P.J. and the University of Wollongong's Faculty of Science, Medicine, and Health's UOW startup grant and Interdisciplinary grant to J.B.D.

## Disclosure

Benefits generated: This research was funded by the New Colombo Plan and the University of Wollongong to build connections between Australian and Fijian universities and researchers. We collaborated with local researchers at The University of the South Pacific (USP) and consulted with/stayed with/hired local people during our field campaign (see Acknowledgements). We returned three Honours theses and additional information to Fijian Ministries, presented at USP as part of their PaCE‐SD seminar series, and brought USP students into the field to learn about bees and our sampling techniques. We additionally left a framed poster to help with tourist engagement at Navai village, featuring *Hylaeus navai*—named in thanks for their ongoing support. This project is aimed at making data and methods available for future work in the Pacific and to build ongoing connections, collaborations, and capacity in the region.

## Ethics Statement

Ethics are not required for insect research as confirmed by the ethics committees at both the University of Wollongong and The University of the South Pacific.

## Conflicts of Interest

The authors declare no conflicts of interest.

## Supporting information


**Figure S1:** Principle components analysis of *H. navai* with assigned geographic populations based on sampling coordinates.
**Figure S2:** Stairway plot reconstruction of effective population size for *H. navai* under different generation time assumptions: (A) one generation per year, (B) two generations per year, (C) four generations per year and (D) six generations per year. The shaded area represents the 95% confidence intervals around the median effective population size estimates. Both axes are plotted on a log scale, with the *y*‐axis showing median effective population size and the *x*‐axis showing approximate calendar year.
**Figure S3:** Cross‐validation scores output from *tess3r* analysis for number of ancestral populations (*K*) for *H. navai* females.
**Figure S4:** Isolation by distance analysis by individual of *H. navai* females.

**Table S1:** Historical effective population size (*N*e) for *H. navai* estimated using a stairway plot analysis, including median effective population size, proportional and percentage change between time intervals, and estimated decline over each 10‐year period.

## Data Availability

Our occurrence data, genetic data (raw and processed), main analysis script, and species distribution modelling and sampling map creation scripts are available on figshare (https://doi.org/10.6084/m9.figshare.33368119). Our new COI sequences can be found under the GenBank accession numbers: PZ291985–PZ292005.
