# Supplementary material for: Genomics Reveal Population Crash and Range Contraction in Elusive Fijian Hylaeus Bees
Source: Ecol Evol. 2026 Oct 5;16(10):e74366. doi: 10.1002/ece3.74366 (PMC13638488; doi:10.1002/ece3.74366)
Supplement: Supplementary file 1 [file ECE3-16-e74366-s001.docx]

**Supporting Information**

Figure S1. Principle components analysis of H. navai with assigned geographic populations based on sampling coordinates.

Figure S2. Stairway plot reconstruction of effective population size for *H. navai* under different generation time assumptions: (A) one generation per year, (B) two generations per year, (C) four generations per year, and (D) six generations per year. The shaded area represents the 95% confidence intervals around the median effective population size estimates. Both axes are plotted on a log scale, with the y-axis showing median effective population size and the x-axis showing approximate calendar year.

Figure S3. Cross-validation scores output from *tess3r* analysis for number of ancestral populations (K) for *H. navai* females.

Figure S4. Isolation by distance analysis by individual of *H. navai* females.

Supplementary table 1. 9.4_G6_dropSheet_g6.xlsx

Historical effective population size (Ne) for *H. navai* estimated using a stairway plot analysis, including median effective population size, proportional and percentage change between time intervals, and estimated decline over each 10-year period.
